# The association between subgroups of MRI findings identified with latent class analysis and low back pain in 40-year-old Danes

**DOI:** 10.1186/s12891-018-1978-x

**Published:** 2018-02-20

**Authors:** Rikke K. Jensen, Peter Kent, Tue S. Jensen, Per Kjaer

**Affiliations:** 1 Institute of Regional Health Research, University of Southern Denmark, Odense, Denmark; 2 Medical Department, Spine Centre of Southern Denmark, Lillebaelt Hospital, Middelfart, Denmark; 3 Nordic Institute of Chiropractic and Clinical Biomechanics, Odense, Denmark; 4 Department of Physiotherapy and Exercise Science, Curtin University, Perth, Australia; 5 Department of Diagnostic Imaging, Silkeborg Hospital, Silkeborg, Denmark; 6 Department of Sports Science and Clinical Biomechanics, University of Southern Denmark, Odense, Denmark

**Keywords:** Low back pain, MRI, Latent class analysis, Subgroups

## Abstract

**Background:**

Research into the clinical importance of spinal MRI findings in patients with low back pain (LBP) has primarily focused on single imaging findings, such as Modic changes or disc degeneration, and found only weak associations with the presence of pain. However, numerous MRI findings almost always co-exist in the lumbar spine and are often present at more than one lumbar level. It is possible that multiple MRI findings are more strongly associated with LBP than single MRI findings. Latent Class Analysis is a statistical method that has recently been tested and found useful for identifying latent classes (subgroups) of MRI findings within multivariable datasets. The purpose of this study was to investigate the association between subgroups of MRI findings and the presence of LBP in people from the general population.

**Methods:**

To identify subgroups of lumbar MRI findings with potential clinical relevance, Latent Class Analysis was initially performed on a clinical dataset of 631 patients seeking care for LBP. Subsequently, 412 participants in a general population cohort (the ‘Backs on Funen’ project) were statistically allocated to those existing subgroups by Latent Class Analysis, matching their MRI findings at a segmental level. The subgroups containing MRI findings from the general population were then organised into hypothetical pathways of degeneration and the association between subgroups in the pathways and the presence of LBP was tested using exact logistic regression.

**Results:**

Six subgroups were identified in the clinical dataset and the data from the general population cohort fitted the subgroups well, with a median posterior probability of 93%–100%. These six subgroups described two pathways of increasing degeneration on upper (L1-L3) and lower (L4-L5) lumbar levels. An association with LBP was found for the subgroups describing severe and multiple degenerative MRI findings at the lower lumbar levels but none of the other subgroups were associated with LBP.

**Conclusion:**

Although MRI findings are common in asymptomatic people and the association between single MRI findings and LBP is often weak, our results suggest that subgroups of multiple and severe lumbar MRI findings have a stronger association with LBP than those with milder degrees of degeneration.

**Electronic supplementary material:**

The online version of this article (10.1186/s12891-018-1978-x) contains supplementary material, which is available to authorized users.

## Background

Disabling low back pain (LBP) profoundly reduces the quality of life of individuals. It also results in a large economic burden for society [1]. But despite the large body of research that has focused on the treatment of LBP, little progress has been made towards identifying best practice management of LBP that leads to substantially better patient outcomes [2]. One reason for this is likely to be the current lack of understanding of the aetiology of most LBP and an inability to readily identify a definitive source of back pain in the majority of individual patients.

Although Magnetic Resonance Imaging (MRI) is often used to try to identify potentially pain causing pathology in the low back, its use remains controversial due to often weak or inconsistent associations with present or future LBP [3, 4]. Although the prevalence of MRI findings is often higher in people with LBP than without, many MRI findings are common in the asymptomatic population [5], making it difficult to associate a single MRI finding with an individual patient’s likely source of pain. For example, the association between spinal degenerative findings and LBP [6].

Furthermore, the association between MRI findings and LBP is often studied for only single MRI findings [7], despite numerous MRI findings commonly being present at the same time. For example, vertebral endplate signal changes (VESC) and disc herniations almost always coexist with other degenerative disc findings, such as reduction in the height and signal intensity of the disc [8, 9]. It has therefore been suggested that combinations of MRI findings could be more strongly associated with LBP than single MRI findings [10].

In a previous study, we investigated ways in which lumbar MRI findings group together as a method to better describe the multivariable relationships between degenerative MRI findings in the lumbar spine [11]. Using Latent Class Analysis, we identified clinically meaningful subgroups of MRI findings [11] and were able to organise these into hypothetical degenerative pathways that were biologically plausible and had face validity. In a subsequent study, this method was found to be reproducible, which suggests that Latent Class Analysis may provide a new approach to investigating the relationship between MRI findings and LBP [12].

The association between MRI findings and the presence of pain can readily be studied in a general population sample because such samples contain both people who have pain and those who do not. This is different from a clinical sample of patients all seeking care for LBP. However, a strength of a clinical sample for deriving and characterising clinically meaningful subgroups of MRI findings is that if there is a relationship between specific MRI subgroups and pain, those subgroups are most likely to be observed in samples of people seeking care and be observable due to an adequate prevalence.

Therefore, the objectives of this study were: 1) to test how well MRI data from people in the general population fit a latent class model that was generated from a clinical population, and 2) to investigate if an association exists between subgroups of lumbar MRI findings organised into degenerative pathways and the presence of LBP in people from the general population.

## Methods

### Design

This study is a secondary analysis of data from two cross-sectional observational studies of two different cohorts.

### Study samples

Two datasets were used for this study: 1) A ‘clinical sample’ of MRI variables and basic demographic data collected in a chronic LBP population, and 2) a dataset with MRI variables and clinical information collected from a ‘general population sample’.

### Clinical sample

To develop a potentially clinically relevant statistical model to analyse the general population data, we used a clinical patient sample collected from June 2006 to June 2008 at an outpatient spine clinic (Spine Centre of Southern Denmark). These were patients with persistent LBP who had been referred from the primary care sector for a multidisciplinary evaluation at this publicly-funded hospital department. The participants in this sample were those who had been considered potential participants for a randomised controlled trial (RCT) [13] due to their meeting the following criteria: (a) LBP or leg pain of at least 3 on an 11-point Numerical Rating Scale, (b) duration of current symptoms from 2 to 12 months, and (c) age above 18 years. All these participants had had a lumbar spine MRI as part of their clinical evaluation. In total, MRI findings and basic demographic data were available on 631 patients, which created a total sample pool of 3155 vertebral motion segments. The mean age of the sample was 42 years (SD 10.8, range 18–73) and 54% were women. Additional details about this sample have previously been published [13].

### General population sample

This study sample consisted of MRI and questionnaire data from a Danish population-based cohort, the ‘Backs on Funen’ project, designed to investigate risk factors associated with LBP, including MRI findings, in a general population [14]. Briefly, the cohort was sampled in June 2000 in the county of Funen, Denmark, which had approximately 500,000 inhabitants. The Central Office of Civil Registration randomly selected every ninth person who was 40 years of age, had been born in Denmark and was living in the county of Funen. The sample was selected to be demographically representative of their age group in Denmark. Potential participants received a letter with an invitation to participate in the study, the procedures of which included an MRI scan of the lumbar spine, a physical spinal examination and completion of questionnaires. In total, 213 women and 199 men aged 40 years of age (*n* = 412) participated in the study.

### MRI

MRI scans in both the general population sample and clinical sample were performed with the same 0.2 T MRI-system (Magnetom Open Viva; Siemens AG, Erlangen, Germany). A body spine surface coil was used for imaging of the lumbar region, with the study subjects in the supine position. Sagittal T1- and T2-weighted and axial T2-weighted MRI images were performed. Further details can be found in the original articles that reported on the general population [14] and clinical [13] samples.

Both MRI datasets were quantitatively coded using a detailed, standardised, research MRI evaluation protocol [15, 16] by the same experienced musculoskeletal research radiologist, who was blinded to any participant information other than name, age and sex. Testing of the MRI evaluation protocol, that included the same radiologist, showed moderate to almost perfect agreement for inter- and intra-observer reliability (kappa range 0.59–1.0) [14–16].

### Variables of interest

To enable transferability of the Latent Class Analysis model across the two samples, only MRI variables common to both samples were selected and some recoding of variables was initially required to harmonise their content. The following 11 MRI variables were selected: Disc signal intensity had three categories: (1) hyper-intense or hyper-intense with visible intra-nuclear cleft, (2) intermediate signal intensity, and (3) hypo-intense; Disc height had four categories: (1) disc higher than the disc above, (2) disc as high as the disc above (if normal), (3) disc narrower than the disc above (if normal) and (4) endplates almost in contact; High Intensity Zone (HIZ) was coded as present or not present; Disc herniation had six categories: (1) no herniation, (2) disc bulge, (3) focal protrusion, (4) broad-based protrusion, (5) extrusion and (6) sequestration; Type of VESC was coded individually for each endplate (upper and lower) at each motion segment and had seven categories: (1) No VESC, (2) Type I, (3) Type II, (4) Type III, (5) Mixed I/II, (6) Mixed II/III, (7) Mixed I/III; Size of VESC was based on its depth of extension into the vertebral body height and had five categories: (1) No VESC, (2) endplate only, (3) > endplate to 25% of the vertebral body height, (4) 25 to 50%, and (5) > 50%; Irregular endplates, local endplate defects and osteophytes were all coded as present or not present. Anterolisthesis was graded according to the Meyerding classification system [17] with four categories. Retrolisthesis was coded as present or not present.

### Low back pain outcomes

In the questionnaire, patients had been asked if they had LBP in the past month, in the past year and if they had sought care during the past year due to their LBP. Furthermore, they were asked if they had experienced LBP for more than 30 days within the last year last year and whether LBP influenced their daily activities and work ability. From these questions, a composite variable ‘non-trivial LBP’ was created, which was defined as LBP for more than 30 days during the last year with at least one consequence: seeking care, reduced time at work, changed work function or reduction in leisure-time activities. Further details about the LBP outcomes have been published elsewhere [18]. All three measures of pain, ‘LBP past month’, ‘LBP past year’ and ‘non-trivial LBP’ were used in the current study.

### Statistical analyses

The latent data structure of subgroups of MRI findings was identified in the clinical sample using multivariable Latent Class Analysis in the statistical program ‘Latent Gold’ (version 4.5, Statistical Innovations, Belmont MA, USA). As an individual’s lumbar motion segments could have varying stages of degeneration, the analyses were made at the level of the vertebral segments, and every participant contributed five lumbar vertebral motion segments to the analysis.

The default settings of the software were used for this analysis and the Latent Class Analysis model was repeated 10 times, because Latent Class Analysis starts with a random starting point that can produce slightly different results. Models with an increasing number of classes were estimated until the best-fitting model was observed. The lowest Bayesian Information Criterion (BIC), which is a method for determining which subgroup solution explains the most variance while requiring the simplest specification of the model, is often used to identify the best model solution. However, in some datasets, the BIC may drop with increasing numbers of subgroups but with ever more small amounts and not reach a ‘floor amount’. Therefore, we adopted the additional criterion of requiring BIC to change at least 1% when further subgroups were added [19].

After choosing the best-fitting latent class model, that model was rerun but with the participants from the general population sample merged with a case weight of 0.001 into the clinical sample dataset. This very low case weighting ensured that the general population participants did not change the latent class model but each of their vertebral segments were classified (with a posterior probability) into the MRI subgroups that best fitted their scoring pattern.

The posterior probability, which is a measure that quantifies the certainty of the membership of a motion segment to a subgroup, was calculated for both samples. Motion segments with high posterior probabilities indicated a good fit to the pattern of the subgroup and low scores indicated a poor fit. Motion segments were assigned to the subgroup where they had the highest posterior probability and the median and range of the posterior probabilities were reported. The clinical sample data were then deleted leaving only the general population sample data. The proportion of vertebral motion segments with MRI findings within each subgroup was calculated and graphed using Excel 2010 (Microsoft Corporation, Redmond, WA, USA). Also, the proportions of vertebral levels (L1/2, L2/3, L3/4 L4/5 and L5/S1) in each subgroup were calculated. Using content analysis, the subgroups were categorised independently by two of the authors (RKJ and TSJ) into hypothetical pathways of degeneration. The subgroup with the least MRI findings was placed at the left side and the remaining subgroups were then organised into one or more pathways with increasing severity and number of findings. This method has previously been tested and been found to show reproducibility [12].

The Latent Class Analysis was performed at the level of the vertebral motion segment using five levels per person, and therefore, a person could potentially belong to five different subgroups. However, when testing the association between subgroup membership and pain, each person needed to belong to a specific subgroup. In previous work [11, 12], Latent Class Analysis had consistently shown a subgroup containing motion segments with no or few MRI findings. So, people with all segmental levels belonging to this subgroup were used as a reference group in the association analysis. The position of a subgroup in the formed pathways was then used to address the issue of assigning subgroup membership at a whole person level, based on the principle that increasingly severe subgroups were to the right side of the pathway diagrams. Therefore, the method used was that a person was allocated to the most right-sided subgroup in which they had one or more vertebral segments.

Each subgroup was considered a separate entity and Exact Logistic Regression was used to test the potential association (Odds Ratios (OR) with 95% confidence intervals (CI)) with each of the three different LBP outcomes as dependent variables and subgroup membership (with a subgroup of no or few MRI findings as the reference) as the independent variable. Exact Logistic Regression was chosen due to a small number of people in some of the contingency tables created from the subgroups. Statistical significance was set as alpha of < 0.05 and STATA 14.0 (StataCorp, College Station, Texas 77,845, USA) was used for analyses and data management.

## Results

### Subgroups of MRI findings and pathways

Latent Class Analysis identified six subgroups of MRI findings for the framework in the clinical sample. The posterior probabilities for the six subgroups had a median range from 99.7% to 100%. The posterior probability for the general population sample that was merged into these six subgroups had a median range from 93% to 100%. More detail is shown in Table 1 and Table 2.

**Table 1:** Posterior probabilities for subgroup membership developed from the clinical sample, *n* = 3155 motion segments

Subgroup number	Median	Inter quartile range	Min.	Max.
1	0.999	0.000	0.677	0.999
2	0.999	0.002	0.519	1.000
3	0.999	0.003	0.395	1.000
4	1.000	0.002	0.534	1.000
5	0.997	0.033	0.602	1.000
6	0.998	0.054	0.537	1.000

**Table 2:** Posterior probability for subgroup membership developed from the general population sample, *n* = 2060 motion segments

Subgroup number	Median	Inter quartile range	Min.	Max.
1	0.997	0.014	0.525	0.997
2	0.996	0.086	0.589	1.000
3	0.991	0.018	0.776	1.000
4	1.000	0.003	0.524	1.000
5	0.925	0.111	0.823	1.000
6	0.994	0.059	0.510	1.000

**Fig. 1:**
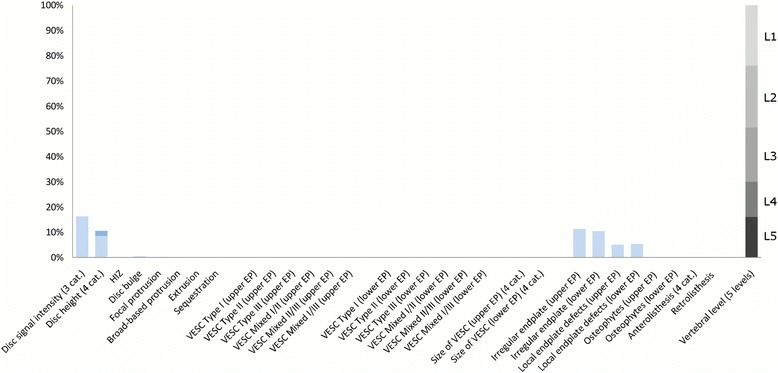
Prevalence of MRI findings in Subgroup 1 of the general population sample. This subgroup contained 65% of the 2060 vertebral motion segments. The vertical bars on the graph represent the proportion of vertebral motion segments for each of the MRI pathologies. The vertebral level indicator shows the relative proportion of vertebral levels (L1 to L5) within the subgroup

**Fig. 2:**
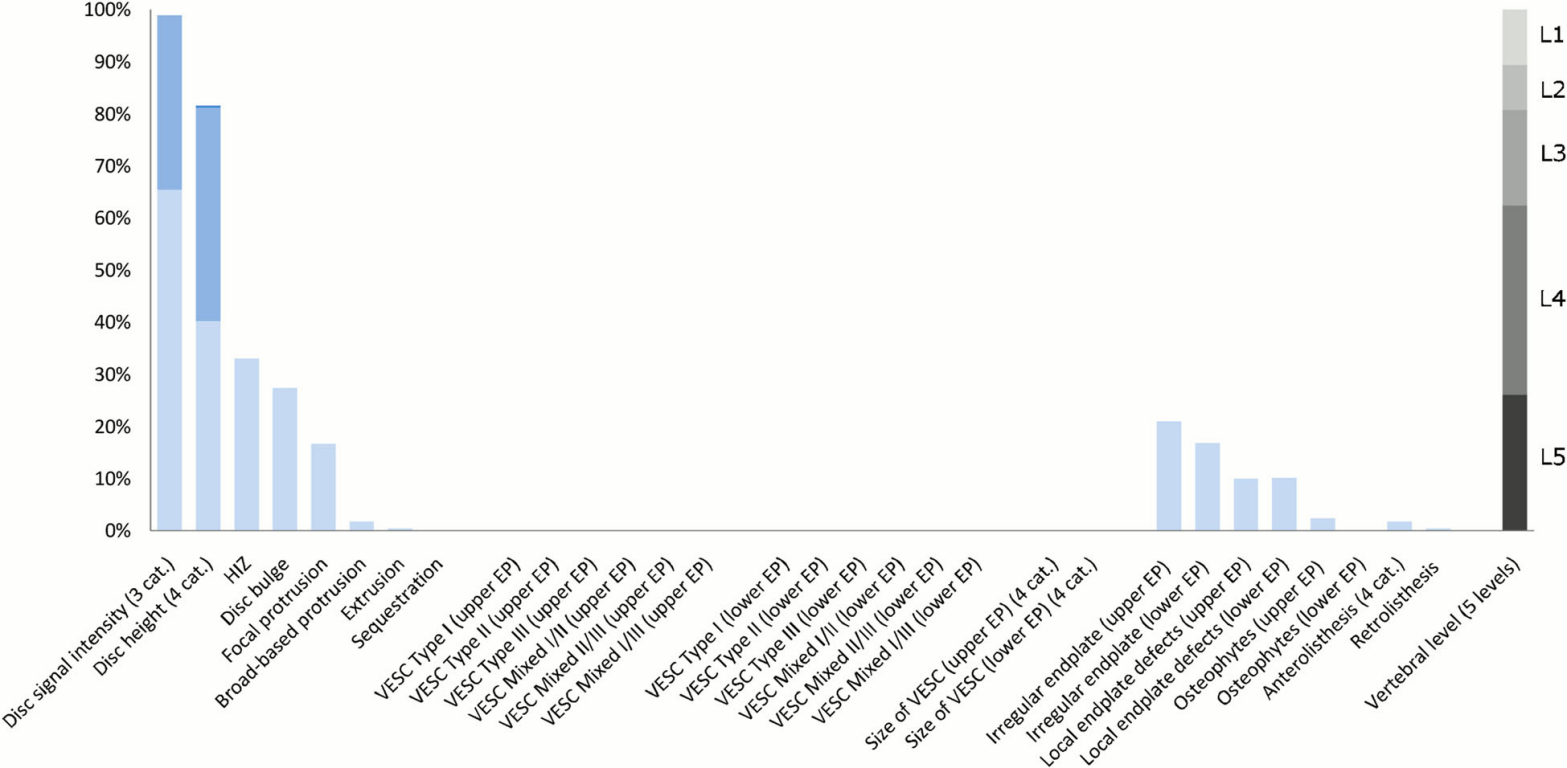
Prevalence of MRI findings in Subgroup 2 of the general population sample. This subgroup contained 22% of the 2060 vertebral motion segments. The vertical bars on the graph represent the proportion of vertebral motion segments for each of the MRI pathologies. The vertebral level indicator shows the relative proportion of vertebral levels (L1 to L5) within the subgroup

**Fig. 3:**
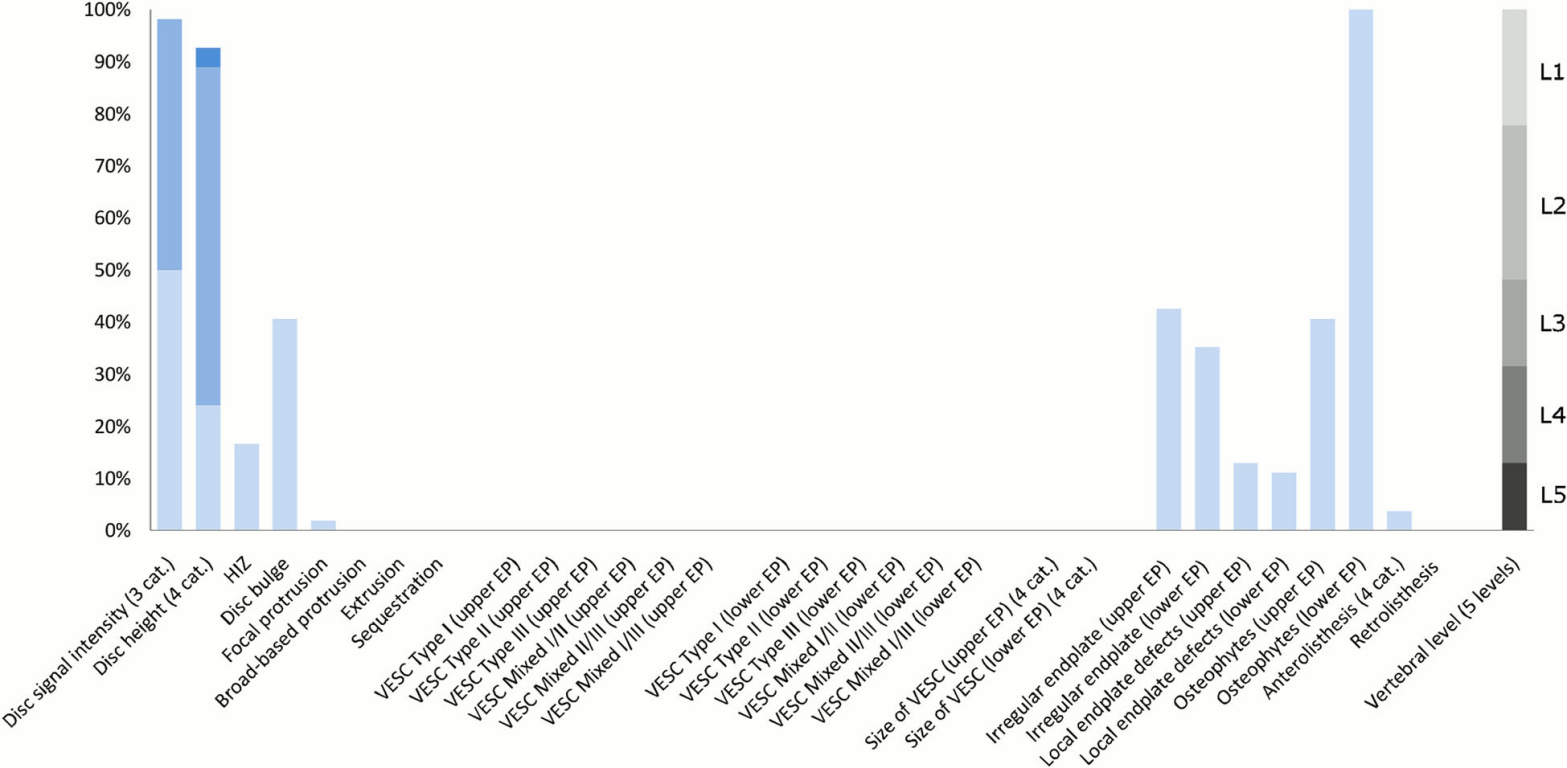
Prevalence of MRI findings in Subgroup 3 of the general population sample. This subgroup contained 3% of the 2060 vertebral motion segments. The vertical bars on the graph represent the proportion of vertebral motion segments for each of the MRI pathologies. The vertebral level indicator shows the relative proportion of vertebral levels (L1 to L5) within the subgroup

**Fig. 4:**
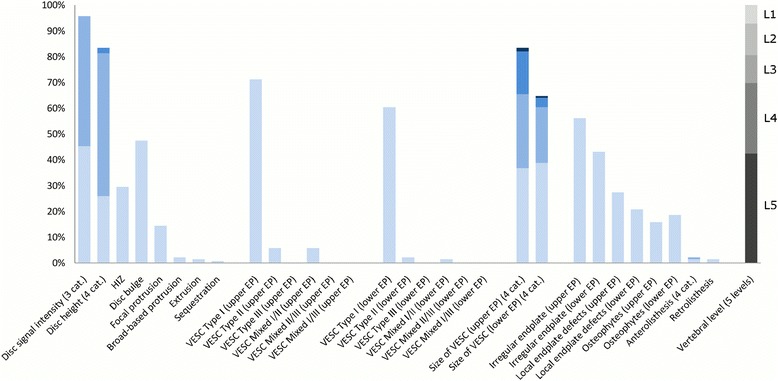
Prevalence of MRI findings in Subgroup 4 of the general population sample. This subgroup contained 7% of the 2060 vertebral motion segments. The vertical bars on the graph represent the proportion of vertebral motion segments for each of the MRI pathologies. The vertebral level indicator shows the relative proportion of vertebral levels (L1 to L5) within the subgroup

**Fig. 5:**
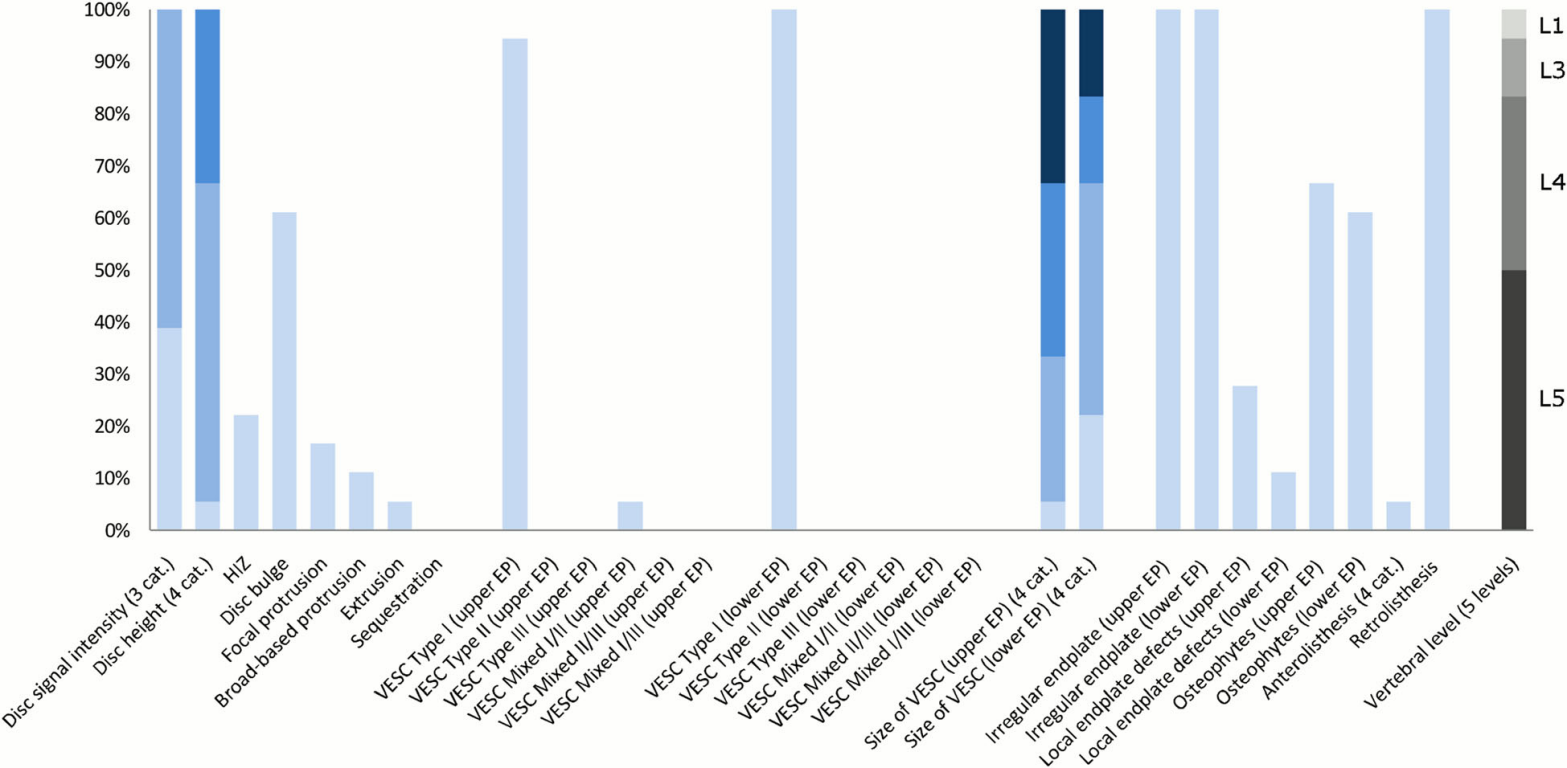
Prevalence of MRI findings in Subgroup 5 of the general population sample. This subgroup contained 1% of the 2060 vertebral motion segments. The vertical bars on the graph represent the proportion of vertebral motion segments for each of the MRI pathologies. The vertebral level indicator shows the relative proportion of vertebral levels (L1 to L5) within the subgroup

**Fig. 6:**
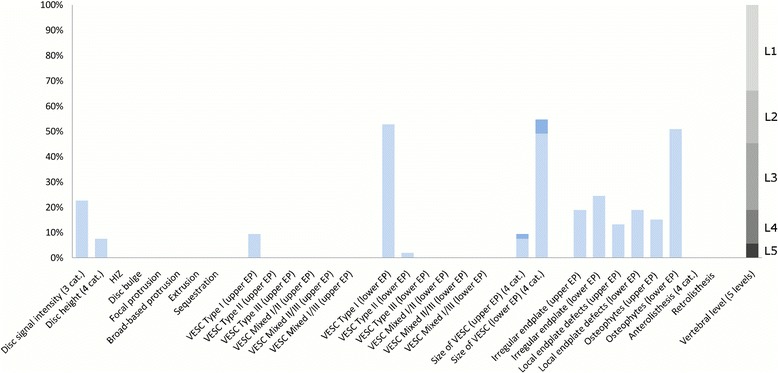
Prevalence of MRI findings in Subgroup 6 of the general population sample. This subgroup contained 3% of the 2060 vertebral motion segments. The vertical bars on the graph represent the proportion of vertebral motion segments for each of the MRI pathologies. The vertebral level indicator shows the relative proportion of vertebral levels (L1 to L5) within the subgroup

The largest subgroup (Subgroup 1) contained 65% of the 2060 lumbar motion segments and represented the normal, pre-degenerative, motion segments (‘No or few findings’) (Fig. 1). Seventy percent of the motion segments in Subgroup 1 were located to the upper lumbar levels (L1, L2 and L3). In contrast, the motion segments in Subgroups 2, 4 and 5 were primarily located at the lower lumbar levels (L4 and L5). Subgroup 2 (‘Mild degeneration - lower vertebral levels’) contained 22% of the motion segments and was characterised by having minor disc degenerative changes, 30% had HIZ and disc protrusions and approximately 20% had focal protrusions, local endplate defects and endplate irregularity (Fig. 2). Subgroup 4 (‘Moderate degeneration and VESC - lower vertebral levels’) contained 7% of the motion segments and was dominated by the findings of moderate disc degeneration, VESC type 1 of moderate size and around half the segments had disc bulges and irregular endplates (Fig. 4). Subgroup 5 (‘Severe degeneration and VESC - lower vertebral levels’) contained 1% of the segments and all had severe disc degeneration, large VESC type 1 and irregular endplates, two thirds had disc bulge and osteophytes, while 10–30% had local endplate defects, HIZ and disc herniations (Fig. 5). Subgroup 3 (‘Mild degeneration, and VESC - upper vertebral levels’) (Fig. 3) and Subgroup 6 (‘Moderate degeneration- upper vertebral levels’) (Fig. 6) both contained 3% of the lumbar segments, primarily located at the upper lumbar levels. In Subgroup 6, approximately 50% of the segments had VESC type 1, mostly at the lower endplates of the motion segments and approximately one third had irregular endplates, local endplate defects and osteophytes together with mildly reduced disc signal intensity. Segments in Subgroup 3 had more severe disc degenerative changes, 40% had disc bulge and irregular endplates, while all the segments had osteophytes.

Each subgroup and its distribution of MRI findings is shown diagrammatically in Figs. 1, 2, 3, 4, 5 and 6.

**Fig. 7:**
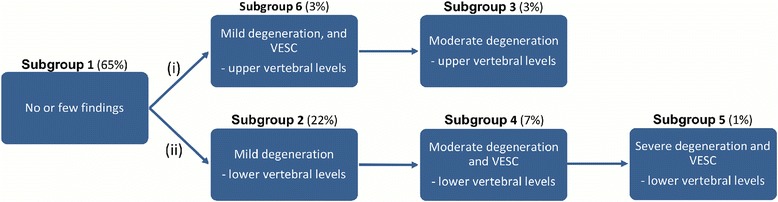
Hypothetical degenerative pathways of the vertebral motion segments. Progressive stages of disc degeneration in the (i) upper lumbar motion segments and (ii) lower lumbar motion segments

Two hypothetical pathoanatomic pathways of degeneration emerged from the content analyses of the subgroups, that describe progressive stages of degeneration in (i) the upper lumbar levels and (ii) the lower lumbar levels. These pathways are illustrated in Fig. 7.

### Association between MRI subgroups and LBP

Seventeen percent (*n* = 72) of the people in the cohort had all their segmental levels in Subgroup 1 and they represent a group of people with no or few MRI findings of vertebral segment degeneration. Therefore, this subgroup was used as the reference group in the logistic regression analysis.

In the first pathway (i) no association was found between people with a lumbar segment belonging to either Subgroup 3 or Subgroup 6 (with no segments in Subgroup 4 or Subgroup 5) and any of the LBP outcomes. Due to a small number of people in some of the pain subgroups, Subgroup 3 and Subgroup 6 were merged and tested for an association with any of the three pain outcomes, but no association was found.

In the second pathway, (ii) the severity of degeneration increased across the subgroups in a plausible direction. Subgroup 5 (Severe degeneration and VESC - lower vertebral levels) contained the segments with the most MRI findings and people with at least one motion segment in Subgroup 5 had an OR of 11.3 (95% CI 1.6–495) for having had LBP the past year and 5.4 (95% CI 1.5–19.6) for having non-trivial LBP compared to those with all 5 levels in Subgroup 1 (No or few findings). People with a minimum of one motion segment in Subgroup 4 (Moderate degeneration and VESC - lower vertebral levels) but no segments in Subgroup 5 had an OR of 3.0 (95% CI 1.5–5.9) for having had LBP in the past year, compared to having all segmental levels in Subgroup 1 (No or few findings). Less than 10 people in Subgroup 5 answered yes to any of the three pain measures and therefore Subgroup 5 was merged with Subgroup 4 [20]. People with a minimum of one motion segment in Subgroup 4 or Subgroup 5 had an OR of 3.4 (95% CI 1.7–7.0) for having had pain within the past year compared to people with all motion segments in Subgroup 1 (No or few findings).

For people who had at least one motion segment in Subgroup 2 (Mild degeneration - lower vertebral levels) and no segments in Subgroups 4 or 5, when compared to people in Subgroup 1, the OR was not significant for any of the outcomes.

**Fig. 8:**
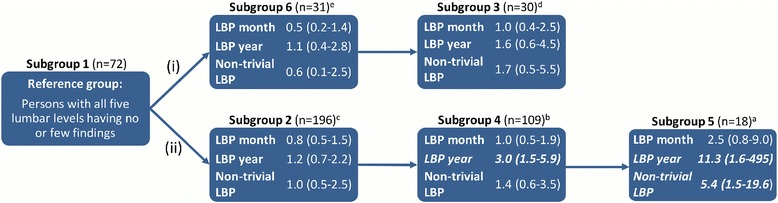
Associations between pain measures and having a motion segment in a specific subgroup. The associations for each of the three pain measures for people having a lumbar motion segment belonging to specific subgroups compared to people in Subgroup 1 (who all had no or few findings in all five motion segments). Results are in Odds Ratios with 95% Confidence Interval. Statistically significant Odds Ratios are italicised and bold. (i) Progressive stages of disc degeneration in the upper lumbar motion segments and (ii) lower lumbar motion segments. ^a^All five motion segments in Subgroup 1 versus a minimum of one motion segment in Subgroup 5. ^b^All five motion segments in Subgroup 1 versus a minimum of one motion segment in Subgroup 4 but no motion segments in Subgroup 5. ^c^All five motion segments in Subgroup 1 versus a minimum of one motion segment in Subgroup 2 but no motion segments in Subgroup 4 or 5. ^d^All five motion segments in Subgroup 1 versus a minimum of one motion segment in Subgroup 3 but no motion segments in Subgroup 4 or 5. ^e^All five motion segments in Subgroup 1 versus a minimum of one motion segment in Subgroup 6 but no motion segments in Subgroup 3, 4 or 5

Results of the logistic regressions are shown in Table 3 and the prevalence of people in the pain subgroups in each subgroup are shown in Additional file 1. Furthermore, the results have been merged with the hypothetical pathoanatomic pathways of degeneration to illustrate the association between pain measures and subgroups (Fig. 8).

**Table 3:** Association between pain measures and belonging to a specific subgroup compared to Subgroup 1

	Odds ratio	95% CI	*p*-value
All five levels in Subgroup 1 versus a minimum of one level in Subgroup 5			
LBP past month	2.5	0.8–9.0	0.15
LBP past year	*11.3*	1.6–495	0.01
Non-trivial LBP	*5.4*	1.5–19.6	0.01
All five levels in Subgroup 1 versus a minimum of one level in Subgroup 4 but no levels in Subgroup 5			
LBP past month	1.0	0.5–1.9	1.00
LBP past year	*3.0*	1.5–5.9	0.00
Non-trivial LBP	1.4	0.6–3.5	0.53
All five levels in Subgroup 1 versus a minimum of one level in Subgroup 4 or 5			
LBP past month	1.1	0.6–2.1	0.82
LBP past year	*3.4*	1.7–7.0	0.00
Non-trivial LBP	1.8	0.8–4.2	0.18
All five levels in Subgroup 1 versus a minimum of one level in Subgroup 2 but no levels in Subgroup 4 or 5			
LBP past month	0.8	0.5–1.5	0.58
LBP past year	1.2	0.7–2.2	0.53
Non-trivial LBP	1.0	0.5–2.5	1.00
All five levels in Subgroup 1 versus a minimum of one level in Subgroup 3 but no levels in Subgroup 4 or 5			
LBP past month	1.0	0.4–2.5	1.00
LBP past year	1.6	0.6–4.5	0.45
Non-trivial LBP	1.7	0.5–5.5	0.48
All five levels in Subgroup 1 versus a minimum of one level in Subgroup 6 but no levels in Subgroup 3, 4 or 5			
LBP past month	0.5	0.2–1.4	0.21
LBP past year	1.1	0.4–2.8	1.00
Non-trivial LBP	0.6	0.1–2.5	0.68
All five levels in Subgroup 1 versus a minimum of one level in Subgroup 3 or 6 but no levels in Subgroup 4 or 5			
LBP past month	0.7	0.3–1.5	0.42
LBP past year	1.3	0.6–2.8	0.61
Non-trivial LBP	1.1	0.4–3.1	1.00

Association between each of the three pain measures for people having a motion segment belonging to specific subgroups compared to people having all five lumbar segments in Subgroup 1 (representing no or few findings). Statistically significant odds ratios are italicised

## Discussion

To our knowledge this is the first study describing how specific subgroups of MRI findings in motion segments, derived using Latent Class Analysis, are associated with pain. Motion segments in Subgroups 4 and 5 had multiple and more severe MRI findings at the lower lumbar levels, and people with these findings were more likely to have had both LBP over the last year and non-trivial LBP compared to those with very few MRI findings on all of their lumbar vertebral levels. None of the other subgroups at the upper or lower lumbar levels were associated with any of the three LBP outcomes. Also, the outcome LBP past month was not associated with any of the subgroups and only moderate consistency was found between the associations with LBP past year and non-trivial LBP.

### Meaning of study and comparison with other studies

In a previous study, the associations between LBP in the past month, LBP in the past year and seeking care for LBP and the single MRI variables in this dataset were analysed by Kjaer et al. [14], who found ORs that were mostly between 1.3 and 2.6 for LBP in the past year and seeking care for LBP. Only reduction in disc signal intensity, reduction in disc height and Modic changes were associated with having LBP in the past month. Modic changes (VESC) were found to be more strongly associated with LBP in the past year (OR 4.2, 95% CI 2.2–8.2) and anterolisthesis (*n* = 12) (OR 6.1, 95% CI 1.0–∞). In our study, both Subgroups 4 and 5 had a large proportion of motion segments with VESC and it is possible that VESC is the single variable that drives the association we observed. On the other hand, VESC could be reflective of a late stage of the degenerative process and therefore VESC could be a proxy for severe degeneration of the lumbar motion segments. This finding is supported by Cheung and colleagues (2009) [21] who found a positive correlation between the sum of degenerative disc MRI findings and LBP that was of more than 2 weeks duration and sufficiently severe to require physician consultation or treatment.

The OR for LBP in the past year (OR 3.4 (95% CI 1.7–7.0)) observed for Subgroup 4 and 5 combined were only moderate in size and therefore that association was no stronger than those previously found for associations with single MRI variables. [14] However, for LBP in the past year the association (OR 11.3 (95% CI 1.6–495)) with Subgroup 5 (Severe degeneration and VESC) was larger than for Subgroup 4 (OR 3.0 (95% CI 1.5–19.6)) (Moderate degeneration and VESC), which suggests a dose-response relationship between severity of MRI findings and risk of symptoms. However, the confidence intervals of the ORs when testing Subgroup 4 and Subgroup 5 independently were very large due to the small cells in the contingency table and therefore this finding should be interpreted with caution.

This finding is in line with that of MacGregor et al. [22], who reported a dose-response relationship between LBP and a sum score [23] of the severity and extend of MRI findings. In their twin study exploring the structural, psychological, and genetic influences on LBP, MacGregor et al. found that structural (MRI) findings were the strongest predictor for LBP and that those with the upper quartile of the sum scores had 3.6 times the odds of reporting pain compared with those with scores in the lowest quartile [22].

These results indicate that spinal pathology or degeneration, as seen on MRI, could still play a role in explaining some of the pain mechanisms in the bio-psycho-social model of LBP. However, as these data also show that 60% of the people with all five lumbar levels in Subgroup 1 (No or few findings) reported pain during the past year, it is obvious that MRI findings do not reflect the only pain mechanism in LBP, which underlines the need for considerable caution when interpreting MRI findings on an individual patient level.

Although, no previous studies have used Latent Class Analysis of vertebral MRI findings to explore their relationship to LBP, others have used Latent Class Analysis in other ways within LBP research. Takatalo et al. [24] investigated the association between LBP severity and lumbar disc degeneration on MRI by using Latent Class Analysis to characterise subgroups of the pain and functional limitation features of 554 young Finnish adults using data collected at age 18, 19 and 21 years. They found five subgroups with different clinical profiles ranging from ‘no pain at all’ to ‘pain at all three time points’. They then related these clinical profiles to single MRI findings and found that disc degeneration and sum scores of disc degeneration were more prevalent in the three most symptomatic pain subgroups compared to the two least symptomatic ones, and that disc degeneration was associated with symptom severity.

Their approach of using Latent Class Analysis to subgroup clinical data, rather than MRI data, ensures that a person only belongs to one subgroup. In contrast, when creating the subgroups in our study, we focused on the co-existence of multiple MRI findings at one segmental level and so every participating person contributed five lumbar vertebral segments to the analysis. However, this approach complicates analysis of the LBP association, as it is the person (not the five vertebral levels) that report the presence of LBP and therefore, a best approximation of allocating an individual person to a single subgroup had to be made, even though this person could have the five vertebral segments in five different subgroups. That said, the modelling approach taken by Takatalo et al. [24] does not ‘solve’ the dilemma of each person having five vertebral segments, it simply does not model this as explicitly and all studies of the association between MRI findings and pain face this same challenge.

### Strengths and weaknesses

The MRI machine used in this study is a low-field (Tesla 0.2) MRI, which generates lower spatial, contrast and temporal resolution than a high-field MRI machine, but this does not automatically lead to lower diagnostic quality. Lee and colleges (2015) found that the diagnostic capability of low-field lumbar spine MRI was very comparable with that of high-field MRI, with Kappa values being excellent for disc herniation and spinal stenosis and good for nerve root compression [25]. Also, in a study by Bendix et al. [26] that compared the investigation of Modic changes (VESC) using low-field MRI (0.3 T) and high-field MRI (1.5 T), they found that these two types of machines were sensitive to different findings, with Modic changes Type 1 being detected three times more often using low-field MRI but Type 2 being detected two times more often when using high-field MRI. This could affect the observed prevalence of VESC in our data samples, although it is unknown which MRI approach is more accurate or if one is just more sensitive and less specific than the other.

A strength of this study is that both datasets were collected specifically for research purposes using the same MRI machine and the same experienced radiologist. However, as data from the two datasets were collected years apart (‘General population sample’ in 2000 and ‘Clinical sample’ in 2006) there is a theoretical risk of the radiologist having changed a cut-point or scale for determining when an MRI finding. This potential bias was addressed by standardising the description of the MRIs by means of a detailed protocol with high reproducibility that was used at both data collections.

The people in the general population cohort were of the same age, ensuring that all lumbar segments have the same ‘chronological age’ and that differences in the degenerative state is reflective of the ‘biological age’ of the motion segment. Although 412 people constituted a fairly large cohort for an imaging study, we still needed to merge two of the subgroups because the prevalence was too low in Subgroup 5 to enable the planned analyses. It is likely that a more detailed model would require an even larger sample size, although the prevalence of these subgroups may vary across settings.

### Perspectives

Latent Class Analysis of MRI data is a novel method and previously we found that this multivariable approach detected clinically meaningful subgroups of MRI findings that could be arranged into biologically plausible hypothetical degenerative pathways with face validity [11]. In a subsequent reproducibility study, we found that using this method in two different MRI samples showed some differences in the content of the subgroups but the overall pattern of increasing degeneration was quite similar [12]. In the current study, we investigated the relationship between MRI findings and the presence of pain over the previous year and found that increasing severity of degeneration was associated with LBP. These results indicate that even though LBP is a multifactorial condition, motion segments with multiple and severe MRI findings could be part of the explanation of LBP. This study was conducted on a cohort of 40-year-old people and as increasing age leads to increasing spinal degeneration, it would be interesting to further study the relationship between subgroups of MRI findings and pain in other age groups, as the relationship between severe degeneration and pain is not necessarily constant over time.

## Conclusion

MRI data from a general population sample fitted the latent class model well, as seen by a typically very high posterior probability for the vertebral segments. The six subgroups of MRI findings represented lumbar vertebral segments with increasing severity of degeneration. Furthermore, our results suggest that multiple and severe lumbar MRI findings of advanced disc degeneration and vertebral endplate signal changes in the lower lumbar spine have a stronger association with pain than milder stages of degeneration.

## Additional file

Additional file 1: The proportion of people in subgroup 1–6 for each pain measure and proportion of people included in each analysis. (PDF 1452 kb)

## Abbreviations

BIC: Bayesian Information Criterion
CI: Confidence Intervals
HIZ: High Intensity Zone
LBP: Low back pain
MRI: Magnetic Resonance Imaging
OR: Odds Ratios
RCT: Randomised Controlled Trial
SD: Standard Deviation
VESC: Vertebral Endplate Signal Changes

## References

[1] Vos T, Flaxman AD, Naghavi M, Lozano R, Michaud C, Ezzati M, Shibuya K, Salomon JA, Abdalla S, Aboyans V (2012). Years lived with disability (YLDs) for 1160 sequelae of 289 diseases and injuries 1990-2010: a systematic analysis for the global burden of disease study 2010. Lancet.

[2] van Middelkoop M, Rubinstein SM, Kuijpers T, Verhagen AP, Ostelo R, Koes BW, van Tulder MW (2011). A systematic review on the effectiveness of physical and rehabilitation interventions for chronic non-specific low back pain. Eur Spine J.

[3] Ract I, Meadeb JM, Mercy G, Cueff F, Husson JL, Guillin R (2015). A review of the value of MRI signs in low back pain. Diagn Interv Imaging.

[4] Steffens D, Hancock MJ, Maher CG, Williams C, Jensen TS, Latimer J (2014). Does magnetic resonance imaging predict future low back pain? A systematic review. Eur J Pain.

[5] Brinjikji W, Diehn FE, Jarvik JG, Carr CM, Kallmes DF, Murad MH, Luetmer PH (2015). MRI findings of disc degeneration are more prevalent in adults with low back pain than in asymptomatic controls: a systematic review and meta-analysis. AJNR Am J Neuroradiol.

[6] Chou D, Samartzis D, Bellabarba C, Patel A, Luk KD, Kisser JM, Skelly AC (2011). Degenerative magnetic resonance imaging changes in patients with chronic low back pain: a systematic review. Spine (Phila Pa 1976).

[7] Videman T, Battie MC, Gibbons LE, Maravilla K, Manninen H, Kaprio J (2003). Associations between back pain history and lumbar MRI findings. Spine (Phila Pa 1976).

[8] Schmid G, Witteler A, Willburger R, Kuhnen C, Jergas M, Koester O (2004). Lumbar disk herniation: correlation of histologic findings with marrow signal intensity changes in vertebral endplates at MR imaging. Radiology.

[9] Wang Y, Videman T, Battie MC (2012). ISSLS prize winner: lumbar vertebral endplate lesions: associations with disc degeneration and back pain history. Spine (Phila Pa 1976).

[10] Endean A, Palmer KT, Coggon D (2011). Potential of magnetic resonance imaging findings to refine case definition for mechanical low back pain in epidemiological studies: a systematic review. Spine (Phila Pa 1976).

[11] Jensen RK, Jensen TS, Kjaer P, Kent P. Can pathoanatomical pathways of degeneration in lumbar motion segments be identified by clustering MRI findings. BMC Musculoskelet Disord. 2013;14:198. 10.1186/1471-2474-14-198. 10.1186/1471-2474-14-198 PMC3706235 23815743

[12] Jensen RK, Kjaer P, Jensen TS, Albert H, Kent P (2016). Degenerative pathways of lumbar motion segments - a comparison in two samples of patients with persistent low back pain. PLoS One.

[13] Jensen RK, Leboeuf-Yde C, Wedderkopp N, Sorensen JS, Manniche C (2012). Rest versus exercise as treatment for patients with low back pain and Modic changes. A randomized controlled clinical trial. BMC Med.

[14] Kjaer P, Leboeuf-Yde C, Korsholm L, Sorensen JS, Bendix T (2005). Magnetic resonance imaging and low back pain in adults: a diagnostic imaging study of 40-year-old men and women. Spine (Phila Pa 1976).

[15] Jensen TS, Sorensen JS, Kjaer P (2007). Intra- and interobserver reproducibility of vertebral endplate signal (modic) changes in the lumbar spine: the Nordic Modic consensus group classification. Acta Radiol.

[16] Solgaard SJ, Kjaer P, Jensen ST, Andersen P (2006). Low-field magnetic resonance imaging of the lumbar spine: reliability of qualitative evaluation of disc and muscle parameters. Acta Radiol.

[17] Meyerding H (1941). Low backage and sciatic pain associated with spondylolisthesis and protruded intervertebral disc. J Bone Joint Surg Am.

[18] Kjaer P, Korsholm L, Leboeuf-Yde C, Hestbaek L, Bendix T (2017). Individual courses of low back pain in adult Danes: a cohort study with 4-year and 8-year follow-up. BMC Musculoskelet Disord.

[19] Kongsted A, Kent P, Hestbaek L, Vach W (2015). Patients with low back pain had distinct clinical course patterns that were typically neither complete recovery nor constant pain. A latent class analysis of longitudinal data. Spine J.

[20] Peduzzi P, Concato J, Kemper E, Holford TR, Feinstein AR (1996). A simulation study of the number of events per variable in logistic regression analysis. J Clin Epidemiol.

[21] Cheung KM, Karppinen J, Chan D, Ho DW, Song YQ, Sham P, Cheah KS, Leong JC, Luk KD (2009). Prevalence and pattern of lumbar magnetic resonance imaging changes in a population study of one thousand forty-three individuals. Spine (Phila Pa 1976).

[22] MacGregor AJ, Andrew T, Sambrook PN, Spector TD (2004). Structural, psychological, and genetic influences on low back and neck pain: a study of adult female twins. Arthritis Rheum.

[23] Sambrook PN, MacGregor AJ, Spector TD (1999). Genetic influences on cervical and lumbar disc degeneration: a magnetic resonance imaging study in twins. Arthritis Rheum.

[24] Takatalo J, Karppinen J, Niinimaki J, Taimela S, Mutanen P, Sequeiros RB, Nayha S, Jarvelin MR, Kyllonen E, Tervonen O (2012). Association of Modic Changes, Schmorl's nodes, Spondylolytic defects, high-intensity zone lesions, disc Herniations, and radial tears with low back symptom severity among young Finnish adults. Spine (Phila Pa 1976).

[25] Lee RK, Griffith JF, Lau YY, Leung JH, Ng AW, Hung EH, Law SW (2015). Diagnostic capability of low- versus high-field magnetic resonance imaging for lumbar degenerative disease. Spine (Phila Pa 1976).

[26] Bendix T, Sorensen JS, Henriksson GA, Bolstad JE, Narvestad EK, Jensen TS (2012). Lumbar modic changes-a comparison between findings at low- and high-field magnetic resonance imaging. Spine (Phila Pa 1976).

