# Supplementary material for: The association between subgroups of MRI findings identified with latent class analysis and low back pain in 40-year-old Danes
Source: BMC Musculoskelet Disord. 2018 Feb 20;19:62. doi: 10.1186/s12891-018-1978-x (PMC5819254; doi:10.1186/s12891-018-1978-x)
Supplement: Supplementary file 1 — The proportion of people in subgroup 1–6 for each pain measure and proportion of people included in each analysis. (PDF 1452 kb) [file 12891_2018_1978_MOESM1_ESM.pdf]

Additional file 1

Table 1 a, b and c: Proportion of people with one or more segmental levels in Subgroup 1 for each of the three pain measures 'LBP past year', 'LBP past month' and 'Non-trivial LBP'

Table 1a

Subgroup 1	No LBP past month; n (%)	LBP past month; n (%)	Total
No	3 (1%)	4 (2%)	7 (2%)
Yes	234 (99%)	171 (98%)	405 (98%)
Total	237	175	412

Table 1b

Subgroup 1	No LBP past year; n (%)	LBP past year; n (%)	Total
No	0 (0%)	7 (2%)	7 (2%)
Yes	128 (100%)	277 (98%)	405 (98%)
Total	128	284	412

Table 1c

Subgroup 1	No non-trivial LBP; n (%)	Non-trivial LBP; n (%)	Total
No	5 (1%)	2 (3%)	7 (2%)
Yes	332 (99%)	73 (97%)	405 (98%)
Total	337	75	412

Table 2 a, b and c: Proportion of people with one or more segmental levels in Subgroup 2 for each of the three pain measures 'LBP past year', 'LBP past month' and 'Non-trivial LBP'

Table 2a

Subgroup 2	No LBP past month; n (%)	LBP past month; n (%)	Total
No	81 (34%)	50 (29%)	131 (32%)
Yes	156 (66%)	125 (71%)	281 (68%)
Total	237	175	412

Table 2b

Subgroup 2	No LBP past year; n (%)	LBP past year; n (%)	Total
No	49 (38%)	82 (29%)	131 (32%)
Yes	79 (62%)	202 (71%)	281 (68%)
Total	128	284	412

Table 2c

Subgroup 2	No non-trivial LBP; n (%)	Non-trivial LBP; n (%)	Total
No	113 (34%)	18 (24%)	131 (32%)
Yes	224 (66%)	57 (76%)	281 (68%)
Total	337	75	412

Additional file 1

Table 3 a, b and c: Proportion of people with one or more segmental levels in Subgroup 3 for each of the three pain measures 'LBP past year', 'LBP past month' and 'Non-trivial LBP'

Table 3a

Subgroup 3	No LBP past month; n (%)	LBP past month; n (%)	Total
No	209 (88%)	157 (90%)	366 (89%)
Yes	28 (12%)	18 (10%)	46 (11%)
Total	237	175	412

Table 3b

Subgroup 3	No LBP past year; n (%)	LBP past year; n (%)	Total
No	114 (89%)	252 (89%)	366 (89%)
Yes	14 (11%)	32 (11%)	46 (11%)
Total	128	284	412

Table 3c

Subgroup 3	No non-trivial LBP; n (%)	Non-trivial LBP; n (%)	Total
No	301 (89%)	65 (87%)	366 (89%)
Yes	36 (11%)	10 (13%)	46 (11%)
Total	337	75	412

Table 4 a, b and c: Proportion of people with one or more segmental levels in Subgroup 4 for each of the three pain measures 'LBP past year', 'LBP past month' and 'Non-trivial LBP'

Table 4a

Subgroup 4	No LBP past month; n (%)	LBP past month; n (%)	Total
No	173 (73%)	127 (73%)	300 (73%)
Yes	64 (27%)	48 (27%)	112 (27%)
Total	237	175	412

Table 4b

Subgroup 4	No LBP past year; n (%)	LBP past year; n (%)	Total
No	107 (84%)	193 (68%)	300 (73%)
Yes	21 (16%)	91 (32%)	112 (27%)
Total	128	284	412

Table 4c

Subgroup 4	No non-trivial LBP; n (%)	Non-trivial LBP; n (%)	Total
No	247 (73%)	53 (71%)	300 (73%)
Yes	90 (27%)	22 (29%)	112 (27%)
Total	337	75	412

Additional file 1

Table 5 a, b and c: Proportion of people with one or more segmental levels in Subgroup 5 for each of the three pain measures 'LBP past year', 'LBP past month' and 'Non-trivial LBP'

Table 5a

Subgroup 5	No LBP past month; n (%)	LBP past month; n (%)	Total
No	231 (97%)	163 (93%)	394 (96%)
Yes	6 (3%)	12 (7%)	18 (4%)
Total	237	175	412

Table 5b

Subgroup 5	No LBP past year; n (%)	LBP past year; n (%)	Total
No	127 (99%)	267 (94%)	394 (96%)
Yes	1 (1%)	17 (6%)	18 (3%)
Total	128	284	412

Table 5c

Subgroup 5	No non-trivial LBP; n (%)	Non-trivial LBP; n (%)	Total
No	328 (97%)	66 (88%)	394 (96%)
Yes	9 (3%)	9 (12%)	18 (4%)
Total	337	75	412

Table 6 a, b and c: Proportion of people with one or more segmental levels in Subgroup 6 for each of the three pain measures 'LBP past year', 'LBP past month' and 'Non-trivial LBP'

Table 6a

Subgroup 6	No LBP past month; n (%)	LBP past month; n (%)	Total
No	207 (87%)	158 (90%)	365 (89%)
Yes	30 (13%)	17 (10%)	47 (11%)
Total	237	175	412

Table 6b

Subgroup 6	No LBP past year; n (%)	LBP past year; n (%)	Total
No	113 (88%)	252 (89%)	365 (89%)
Yes	15 (12%)	32 (11%)	47 (11%)
Total	128	284	412

Table 6c

Subgroup 6	No non-trivial LBP; n (%)	Non-trivial LBP; n (%)	Total
No	297 (88%)	68 (91%)	365 (89%)
Yes	40 (12%)	7 (9%)	47 (11%)
Total	337	75	412

Additional file 1

Table 7a, b and c: Proportion of people with all segments in Subgroup 1 vs. one or more segments in Subgroup 5 for each of the three pain measures ‘LBP past year’, ‘LBP past month’ and ‘Non-trivial LBP’

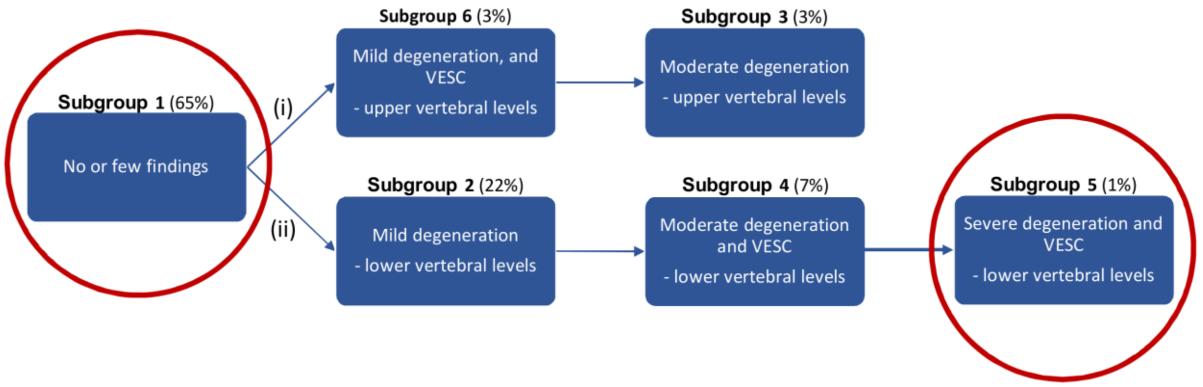

Table 7a

All 5 levels in Subgroup 1 vs min. one level in Subgroup 5	No LBP past month; n (%)	LBP past month; n (%)	Total
Subgroup 1	40 (87%)	32 (73%)	72 (80%)
Subgroup 5	6 (13%)	12 (27%)	18 (20%)
All	46	44	90

Table 7b

All 5 levels in Subgroup 1 vs min. one level in Subgroup 5	No LBP past year; n (%)	LBP past year; n (%)	Total
Subgroup 1	29 (97%)	43 (72%)	72 (80%)
Subgroup 5	1 (3%)	17 (28%)	18 (20%)
All	46	44	90

Table 7c

All 5 levels in Subgroup 1 vs min. one level in Subgroup 5	No non-trivial LBP; n (%)	Non-trivial LBP; n (%)	Total
Subgroup 1	61 (87%)	11 (55%)	72 (80%)
Subgroup 5	9 (13%)	9 (45%)	18 (20%)
All	70	20	90

Additional file 1

Table 8a, b and c: Proportion of people with all segments in Subgroup 1 vs. one or more segments in Subgroup 4 but no segments in Subgroup 5 for each of the three pain measures 'LBP past year', 'LBP past month' and 'Non-trivial LBP'

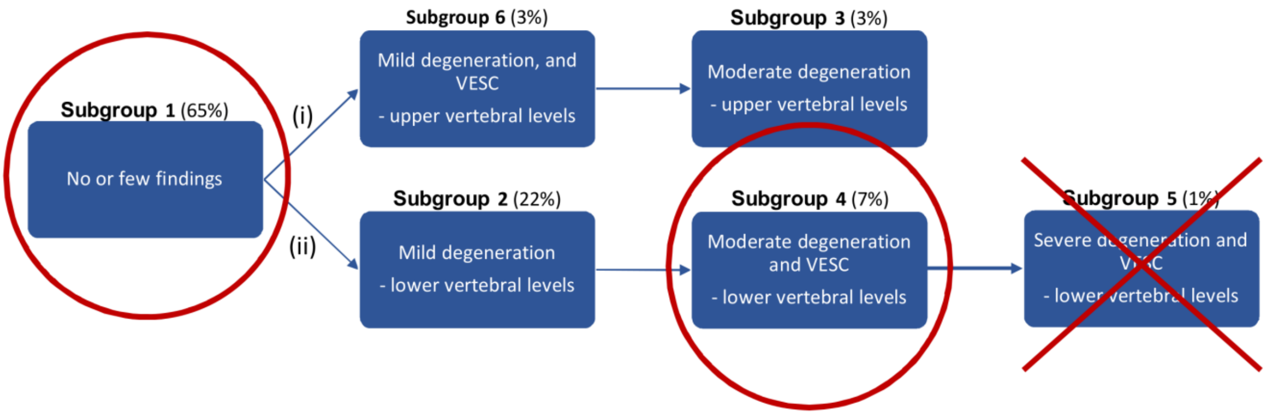

Table 8a

All 5 levels in Subgroup 1 vs min. one level in Subgroup 4 but not Subgroup 5	No LBP past month; n (%)	LBP past month; n (%)	Total
Subgroup 1	40 (40%)	32 (40%)	72 (40%)
Subgroup 4	61 (60%)	48 (60%)	109 (60%)
All	101	80	181

Table 8b

All 5 levels in Subgroup 1 vs min. one level in Subgroup 4 but not Subgroup 5	No LBP past year; n (%)	LBP past year; n (%)	Total
Subgroup 1	29 (59%)	43 (33%)	72 (40%)
Subgroup 4	20 (41%)	89 (67%)	109 (60%)
All	49	132	181

Table 8c

All 5 levels in Subgroup 1 vs min. one level in Subgroup 4 but not Subgroup 5	No non-trivial LBP; n (%)	Non-trivial LBP; n (%)	Total
Subgroup 1	61 (41%)	11 (33%)	72 (40%)
Subgroup 4	87 (59%)	22 (67%)	109 (60%)
All	148	33	181

Additional file 1

Table 9a, b and c: Proportion of people with all segments in Subgroup 1 vs. one or more segments in Subgroup 4 or Subgroup 5 for each of the three pain measures ‘LBP past year’, ‘LBP past month’ and ‘Non-trivial LBP’

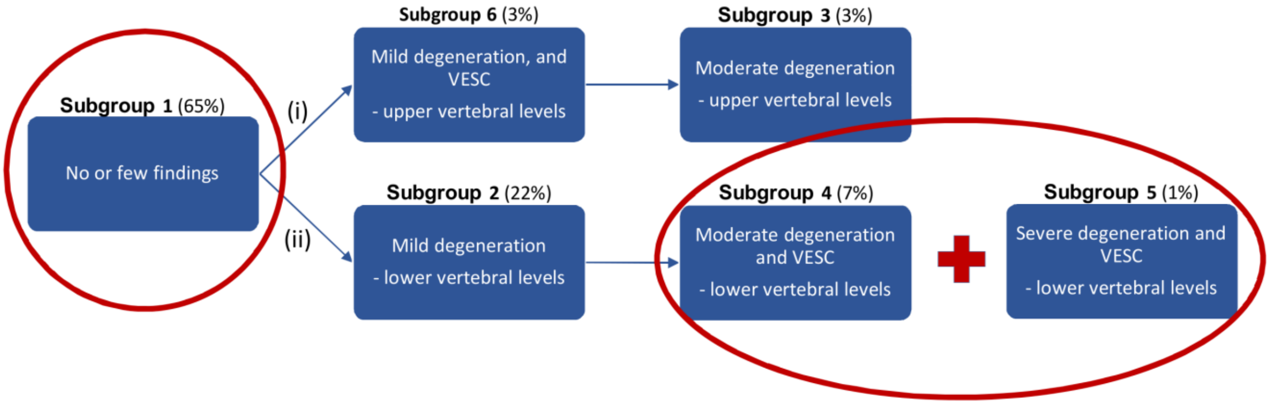

Table 9a

All 5 levels in Subgroup 1 vs min. one level in Subgroup 4 or in Subgroup 5	No LBP past month; n (%)	LBP past month; n (%)	Total
Subgroup 1	40 (37%)	32 (35%)	72 (36%)
Subgroup 4 or 5	67 (63%)	60 (65%)	127 (64%)
All	107	92	199

Table 9b

All 5 levels in Subgroup 1 vs min. one level in Subgroup 4 or in Subgroup 5	No LBP past year; n (%)	LBP past year; n (%)	Total
Subgroup 1	29 (58%)	43 (29%)	72 (36%)
Subgroup 4 or 5	21 (42%)	106 (71%)	127 (64%)
All	50	149	199

Table 9c

All 5 levels in Subgroup 1 vs min. one level in Subgroup 4 or in Subgroup 5	No non-trivial LBP; n (%)	Non-trivial LBP; n (%)	Total
Subgroup 1	61 (39%)	11 (26%)	72 (36%)
Subgroup 4 or 5	96 (61%)	31 (74%)	127 (64%)
All	157	42	199

Additional file 1

Table 10a, b and c: Proportion of people with all segments in Subgroup 1 vs. one or more segments in Subgroup 2 but no segments in Subgroup 4 or Subgroup 5 for each of the three pain measures ‘LBP past year’, ‘LBP past month’ and ‘Non-trivial LBP’

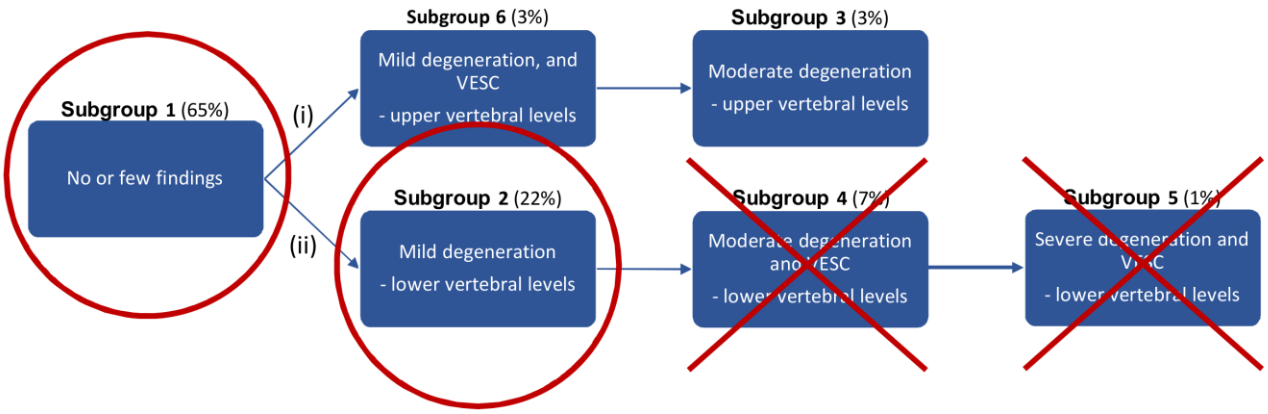

Table 10a

All 5 levels in Subgroup 1 vs min. one level in Subgroup 2 but no levels in Subgroup 4 or Subgroup 5	No LBP past month; n (%)	LBP past month; n (%)	Total
Subgroup 1	40 (25%)	32 (29%)	72 (27%)
Subgroup 2	118 (75%)	78 (71%)	196 (73%)
All	158	110	268

Table 10b

All 5 levels in Subgroup 1 vs min. one level in Subgroup 2 but no levels in Subgroup 4 or Subgroup 5	No LBP past year; n (%)	LBP past year; n (%)	Total
Subgroup 1	29 (30%)	43 (25%)	72 (27%)
Subgroup 2	69 (70%)	127 (75%)	196 (73%)
All	98	170	268

Table 10c

All 5 levels in Subgroup 1 vs min. one level in Subgroup 2 but no levels in Subgroup 4 or Subgroup 5	No non-trivial LBP; n (%)	Non-trivial LBP; n (%)	Total
Subgroup 1	61 (27%)	11 (26%)	72 (27%)
Subgroup 2	165 (73%)	31 (74%)	196 (73%)
All	226	42	268

Additional file 1

Table 11a, b and c: Proportion of people with all segments in Subgroup 1 vs. one or more segments in Subgroup 3 but no segments in Subgroup 4 or Subgroup 5 for each of the three pain measures 'LBP past year', 'LBP past month' and 'Non-trivial LBP'

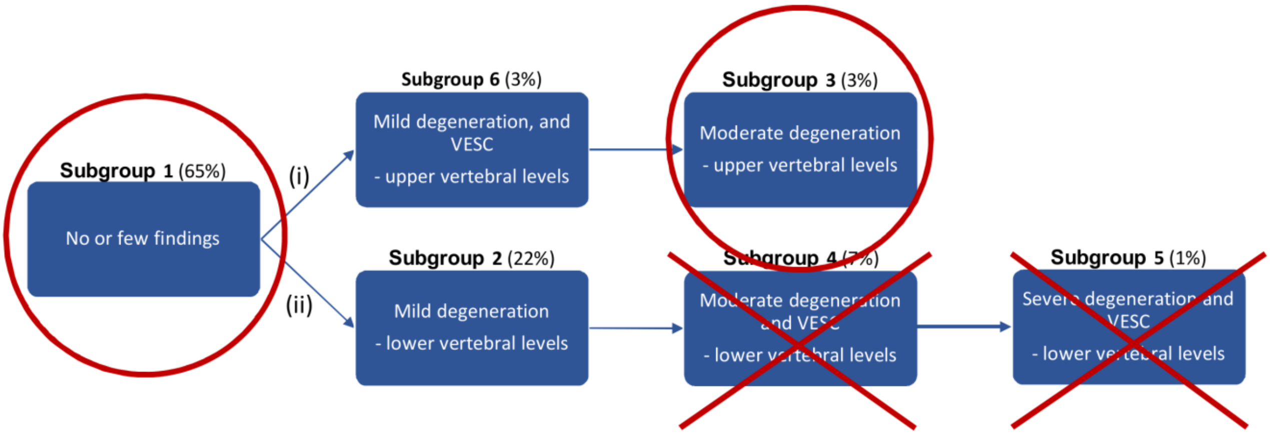

Table 11a

All 5 levels in Subgroup 1 vs min. one level in Subgroup 3 but no levels in Subgroup 4 or 5	No LBP past month; n (%)	LBP past month; n (%)	Total
Subgroup 1	40 (70%)	32 (71%)	72 (71%)
Subgroup 3	17 (30%)	13 (29%)	30 (29%)
All	57	45	102

Table 11b

All 5 levels in Subgroup 1 vs min. one level in Subgroup 3 but no levels in Subgroup 4 or 5	No LBP past year; n (%)	LBP past year; n (%)	Total
Subgroup 1	29 (76%)	43 (67%)	72 (71%)
Subgroup 3	9 (24%)	21 (33%)	30 (29%)
All	38	64	102

Table 11c

All 5 levels in Subgroup 1 vs min. one level in Subgroup 3 but no levels in Subgroup 4 or 5	No Non-trivial LBP n (%)	LBP non-trival; n (%)	Total
Subgroup 1	61 (73%)	11 (61%)	72 (71%)
Subgroup 3	23 (27%)	7 (39%)	30 (29%)
All	84	18	102

Additional file 1

Table 12a, b and c: Proportion of people with all segments in Subgroup 1 vs. one or more segments in Subgroup 6 but no segments in Subgroup 3, Subgroup 4 or Subgroup 5 for each of the three pain measures ‘LBP past year’, ‘LBP past month’ and ‘Non-trivial LBP’

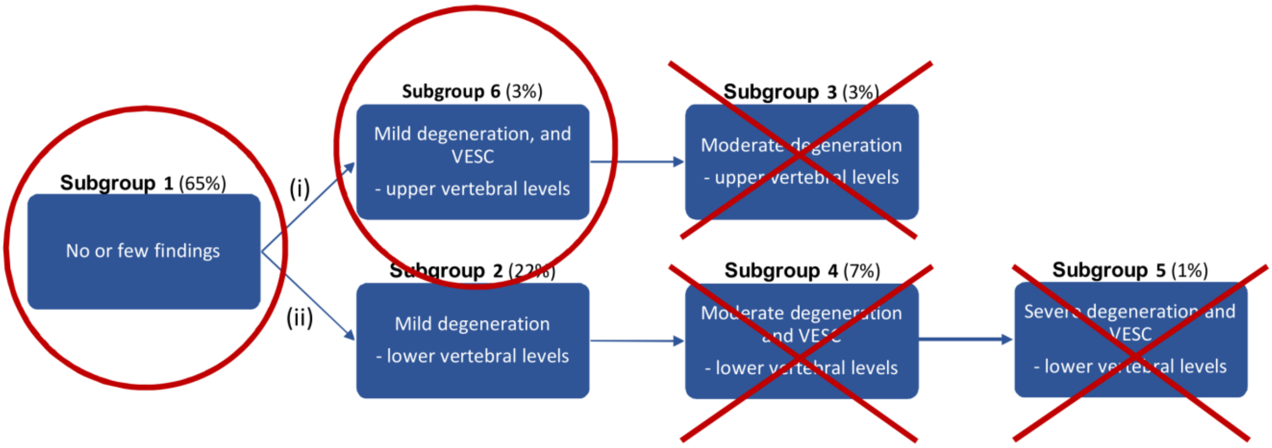

Table 12a

All 5 levels in Subgroup 1 vs min. one level in Subgroup 6 but no levels in Subgroup 4, 5 or 3	No LBP past month; n (%)	LBP past month; n (%)	Total
Subgroup 1	40 (65%)	32 (78%)	72 (70%)
Subgroup 6	22 (35%)	9 (22%)	31 (30%)
All	62	41	103

Table 12b

All 5 levels in Subgroup 1 vs min. one level in Subgroup 6 but no levels in Subgroup 4, 5 or 3	No LBP past year; n (%)	LBP past year; n (%)	Total
Subgroup 1	29 (71%)	43 (69%)	72 (70%)
Subgroup 6	12 (29%)	19 (31%)	31 (30%)
All	41	62	103

Table 12c

All 5 levels in Subgroup 1 vs min. one level in Subgroup 6 but no levels in Subgroup 4, 5 or 3	No non-trivial LBP; n (%)	Non-trivial LBP; n (%)	Total
Subgroup 1	61 (69%)	11 (79%)	72 (70%)
Subgroup 6	28 (31%)	3 (21%)	31 (30%)
All	89	14	103

Additional file 1

Table 13a, b and c: Proportion of people with all segments in Subgroup 1 vs. one or more segments in Subgroup 6 and Subgroup 3 but no segments in Subgroup 4 or Subgroup 5 for each of the three pain measures 'LBP past year', 'LBP past month' and 'Non-trivial LBP'

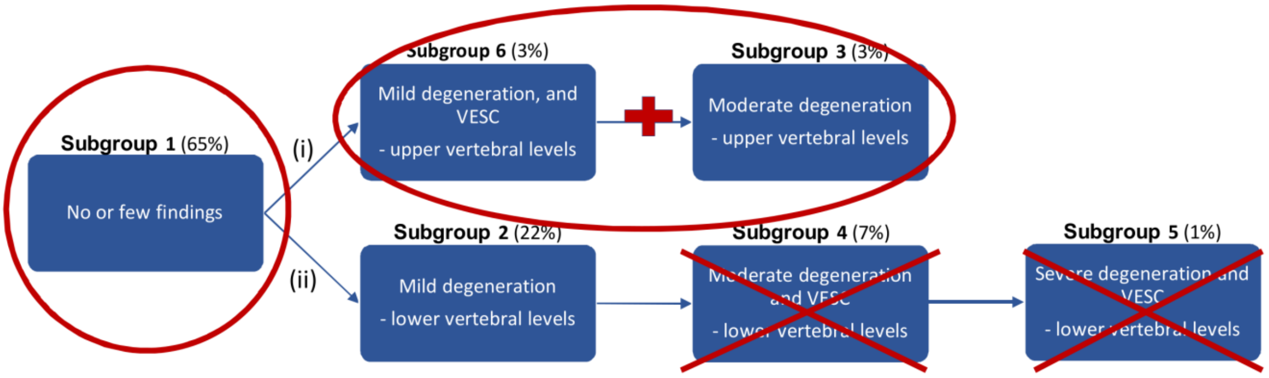

Table 13a

All 5 levels in Subgroup 1 vs min. one level in Subgroup 3 or 6 but no levels in Subgroup 4 or 5	No LBP past month; n (%)	LBP past month; n (%)	Total
Subgroup 1	40 (51%)	32 (59%)	72 (54%)
Subgroup 3 or 6	39 (49%)	22 (41%)	61 (46%)
All	79	54	133

Table 13b

All 5 levels in Subgroup 1 vs min. one level in Subgroup 3 or 6 but no levels in Subgroup 4 or 5	No LBP past year; n (%)	LBP past year; n (%)	Total
Subgroup 1	29 (58%)	43 (52%)	72 (54%)
Subgroup 3 or 6	21 (42%)	40 (48%)	61 (46%)
All	50	83	133

Table 13c

All 5 levels in Subgroup 1 vs min. one level in Subgroup 3 or 6 but no levels in Subgroup 4 or 5	No non-trivial LBP; n (%)	Non-trivial LBP; n (%)	Total
Subgroup 1	61 (54%)	11 (52%)	72 (54%)
Subgroup 3 or 6	51 (46%)	10 (48%)	61 (46%)
All	112	21	133
